# The Surgical Uses of the Hair Pin

**Published:** 1902-10

**Authors:** 


### The Surgical Uses of the Hair Pin.

J. Torrance Rugh, in *American Medicine,* has an interesting and ingenious *résumé* of the application of this common article in emergency work. He divides his subject into three parts. (1) When used in its natural shape. (2) When straightened, and (3) when bent or twisted. He proves that this is an instrument of almost protean utility, being at once pin, suture, probe, curette, female catheter, nasal speculum and aneurism needle; its scope of usefulness seems to be only limited by the individual ingenuity of the surgeon, while it is easily sterilized and always available.

